# Estrogen signaling effects on muscle-specific immune responses through controlling the recruitment and function of macrophages and T cells

**DOI:** 10.1186/s13395-019-0205-2

**Published:** 2019-07-29

**Authors:** Zhao Hong Liao, Tao Huang, Jiang Wei Xiao, Rui Cai Gu, Jun Ouyang, Gang Wu, Hua Liao

**Affiliations:** 1 Guangdong Provincial Key Laboratory of Medical Biomechanics, Department of Anatomy, Southern Medical University, Guangzhou, 510515 China; 2 Department of Emergency, NanFang Hospital, Southern Medical University, Guangzhou, 510515 China

**Keywords:** Estrogen, Myoinjury, Inflammation, Estradiol (E_2_), C_2_C_12_ cell

## Abstract

**Background:**

Estrogen signaling is indispensable for muscle regeneration, yet the role of estrogen in the development of muscle inflammation, especially in the intramuscular T cell response, and the influence on the intrinsic immuno-behaviors of myofibers remain largely unknown. We investigated this issue using the mice model of cardiotoxin (CTX)-induced myoinjury, with or without estrogen level adjustment.

**Methods:**

CTX injection i.m. (tibialis anterior, TA) was performed for preparing mice myoinjury model. Injection s.c. of 17β-estradiol (E_2_) or estrogen receptor antagonist 4-OHT, or ovariectomy (OVX), was used to change estrogen level of animal models in vivo. Serum E_2_ level was evaluated by ELISA. Gene levels of estrogen receptor (ERs) and cytokines/chemokines in inflamed muscle were monitored by qPCR. Inflammatory infiltration was observed by immunofluorescence. Macrophage and T cell phenotypes were analyzed by FACS. Immunoblotting was used to assess protein levels of ERs and immunomolecules in C_2_C_12_ myotubes treated with E_2_ or 4-OHT, in the presence of IFN-γ.

**Results:**

We monitored the increased serum E_2_ level and the upregulated ERβ in regenerated myofibres after myotrauma. The absence of estrogen in vivo resulted in the more severe muscle inflammatory infiltration, involving the recruitment of monocyte/macrophage and CD4^+^ T cells, and the heightened proinflammatory (M1) macrophage. Moreover, estrogen signaling loss led to Treg cells infiltration decrease, Th1 response elevation in inflamed muscle, and the markedly expression upregulation of immunomolecules in IFN-γ-stimulated C_2_C_12_ myotubes in vitro.

**Conclusion:**

Our data suggest that estrogen is a positive intervention factor for muscle inflammatory response, through its effects on controlling intramuscular infiltration and phenotypes of monocytes/macrophages, on affecting accumulation and function of Treg cells, and on suppressing Th1 response in inflamed muscle. Our findings also imply an inhibition effect of estrogen on the intrinsic immune behaviors of muscle cells.

## Background

Estrogen play an essential role in regulating immune response, through its interactions with the receptors on the immune cells which affects the production, maturation, differentiation, and ultimately the functioning of immune cells [1–3]. Generally, estrogen stimulates the production of immunoglobulins by plasma cells [4], and directly upregulates the expression of mediators of B cell survival, such as CD22, SHP-1, and Bcl-2 and impairs mediators of B cell apoptosis such as PD-1 [5–8]. On the other hand, estrogen exerts repressive effects on the innate immune, by increasing regulatory T cells (Tregs) frequency and number [9], controlling the expression of certain chemokine receptors in T cells [10], repressing monocytes and neutrophils to secrete proinflammatory cytokines in response to activating stimuli [11–13], or impairing natural killer (NK) cell cytotoxicity [14]. Taken together, these findings suggest that estrogen signaling is important in establishing the balance of immunity and tolerance.

An important and persistent finding has been that males and females respond differently following traumatic injury, with a relative protection afforded to females [15, 16]. After acute trauma, an early elevation of estrogen levels had been reported [17–19]. Our previous work evidenced that the alterations in the serum estradiol (E_2_) levels is correlated with the severity of trauma and craniocerebral injury and, thus probably, could serve as an indicator for the evaluation of the trauma severity and possibly in prognosis [20]. The evidence from animal models also revealed that estrogen might have an important role in injury healing by directly modifying the local inflammatory and immune reaction. For example, estrogen was reported to (i) reduce the number of wound neutrophils and diminish neutrophil localization at sites of inflammation [21], and (ii) augment estrogen receptor activity in immune cells and dampen innate immune signaling pathways in peripheral dendritic cells and macrophages [22–24]. But anyway, it is still incompletely delineated how the estrogen milieu effects on the immune response trajectory of the damaged peripheral tissue soon after injury.

Acute trauma always involves in skeletal muscle injury, triggers the severe muscle inflammation, swelling, and muscle fiber degeneration [25, 26]. Ideal muscle regeneration and repair is favorable for recovering movement function and improving life quality of trauma patients. Skeletal muscle is a target tissue for estrogen, which expresses both ERs (ERα and ERβ) [27, 28], and in mice ERβ is the predominant isoform [28]. E_2_ is suggested to promote proliferation and differentiation of skeletal muscle myoblasts which express functional estrogen receptors [29]. Estrogen signaling is indispensable for muscle regenerative processes, since this hormone mediates protein synthesis and satellite cell activation, and regulates metabolic homeostasis and insulin sensitivity in skeletal muscle [30, 31]. However, whether in vivo estrogen alteration after acute muscle injury is directly associated with muscle inflammation procedure, especially with intramuscular T cell response, and even associated with the intrinsic immuno-behaviors of myofibres remain largely unknown.

In this study, we explored the role of estrogen signaling on mice acute muscle inflammatory response induced by cardiotoxin (CTX) injection. We monitored the increased serum E_2_ level in mice suffered from myoinjury, and the upregulated ERβ in regenerated myofibres in damaged tibialis anterior (TA) muscle. By in vivo administration of the recombinant 17β-estradiol, estrogen receptor antagonist 4-OHT, or using ovariectomy (OVX) model of female mice, we artificially elevated or blocked serum E_2_ level in vivo. We showed that estrogen signaling contributed to control intramuscular infiltration of monocytes/macrophages, and the transition of proinflammatory M1 to the anti-inflammatory M2 macrophages. Moreover, we found that estrogen signaling affected on accumulation and function of Treg cells, suppresses Th1 response in damaged muscle, and regulates the intrinsic immune capacities of muscle cells. Combined, our data demonstrate an essential role for estrogen signaling in suppression of the acute damage triggered-muscle inflammation and immune response.

## Methods

### Mouse strains

C57BL/6 (B6) mice were obtained from Animal Experimentation Centre of the Southern Medical University. Mice were housed in a specific pathogen-free barrier facility and were analyzed between 8 and 16 weeks of age. Animal experiments were approved by the local institutional ethic committee for animal experimentation.

### Animal experiments

For preparing CTX-induced myoinjury, mice were injected 30 μl CTX (30 μg/ml, Sigma-Aldrich, USA) into the TA muscles. To interfere with estrogen level in vivo, we used the recombinant β-estradiol (E_2_, E2758, Sigma-Aldrich, USA), or estrogen receptor antagonist, 4-Hydroxytamoxifen (4-OHT, T176, Sigma-Aldrich, USA). One day after CTX injection, animals received E_2_ (50 μl/mouse/day, 50 μg/ml) or 4-OHT (50 μl/mouse/day, 2 mg/ml) daily subcutaneous injection (s.c.), respectively [32, 33]. For preparing ovariectomy (OVX) mice, female mice (8w) were kept under anesthesia by intraperitoneal (i.p.) administration of ketamine (90 mg/kg) and xylazine (4.5 mg/kg). A ventral incision between two and three nipples on each side was made and then ovaries were removed slowly. After closing the wound, 22,000 IU/kg penicillin was injected. The sham group underwent the same procedure without removal of the ovaries. Plasma E_2_ level of treated mice were detected by ELISA analysis. OVX mice further received CTX injection into TA muscles on day 14 post-ovariectomy. Mice were sacrificed on day 0, 1, 3, 7, 10, 15, or 20 after myoinjury. TA muscle specimens were collected and snap frozen for gene and protein analysis, or homogenized for cell sorting. For histology, muscle samples were directly frozen in liquid nitrogen-cooled isopentane. For assessing macrophages phagocytosis capacity, female OVX mice or untreated control mice further received 50 μl Lumispheres (Lum, 1:200, 5 mg/ml, BeiSiLe, China) injection into TA muscles 1 day after CTX injection. Mice were sacrificed on day 3, and analyzed by flow cytometry.

### RNA preparation and qRT-PCR

After cervical dislocation, bilateral TA muscles were rapidly dissected out and dipped immediately into RNAlater (RNeasy Protect Mini Kit, Qiagen) to protect RNA from degradation. The muscles were weighted and incubated overnight at 4 °C, then transferred to TRIzol and deep freezed at − 80 °C prior to further analysis.

One microgram of total RNA samples from muscle tissue or C_2_C_12_ myotubes was used for reverse transcription (RT) with commercially available kit (RevertAid First Strand cDNA Synthesis Kit, Fermentas). Real-time polymerase chain reaction (PCR) was performed in triplicate with an ABI StepOne Plus system (Applied Biosystems, USA) and a fluorescence-labeled SYBR Green/ROX qPCRMaster Mix kit (Fermentas) for IL-1, IL-6, IL-10, TGF-β, MCP-1, MIP-1α, SLPI, TNF-α, TGF-β2, PPARγ, IL-1β, IFN-γ, IL-4, IL-17, T-bet, IRF4, STAT3, ERα, and ERβ and with glyceraldehyde-3-phosphate dehydrogenase (GAPDH) taken as an endogenous control (primer sequences and sizes of amplicons are listed in Table 1). The results were analyzed with SOS2.1 software (Applied Biosystems). Expression of the genes was calculated from the accurate threshold cycle (Ct). The Ct values for GAPDH were compared with those from IL-1, IL-6, IL-10, TGF-β, MCP-1, MIP-1α, SLPI, TNF-α, TGF-β2, PPARγ, IL-1β, IFN-γ, IL-4, IL-17, T-bet, IRF4, STAT3, ERα, ERβ, and in each well to calculate ΔCt. Data of the treated conditions were expressed relative to the signal obtained for the average of the untreated controls by the ΔΔCt calculation. The triplicate ΔΔCt values for each sample were averaged.

**Table 1:** Primer sequences for qRT-PCR

Genes	Primer sequence
IL-1	Forward(5′-3′): GCCCATCCTCTGTGACTC
	Reverse(3′-5′): TGTGCCGTCTTTCATTAC
IL-6	Forward(5′-3′): GGCAATTCTGATTGTATG
	Reverse(3′-5′): CTCTGGCTTTGTCTTTCT
MCP-1	Forward(5′-3′): GGGTCCAGACATACATTAA
	Reverse(3′-5′): ACGGGTCAACTTCACATT
IL-10	Forward(5′-3′): TTTCAAACAAAGGACCAG
	Reverse(3′-5′): GGATCATTTCCGATAAGG
MIP-1α	Forward(5′-3′): CTGCCCTTGCTGTTCTTC
	Reverse(3′-5′): CAAAGGCTGCTGGTTTCA
TGF-β	Forward(5′-3′): GGCGGTGCTCGCTTTGTA
	Reverse(3′-5′): TCCCGAATGTCTGACGTATTGA
SLPI	Forward(5′-3′): AAGCCACAATGCCGTACTGACTG
	Reverse(3′-5′): ACAGGATTCACGCACTTGGAACC
TNF-α	Forward(5′-3′): GGCGGTGCCTATGTCTCA
	Reverse(3′-5′): CCTCCACTTGGTGGTTTGT
TGF-β2	Forward(5′-3′): GGCGGTGCTCGCTTTGTA
	Reverse(3′-5′): TCCCGAATGTCTGACGTATTGA
PPARγ	Forward(5′-3′): CGCCAAGGTGCTCCAGAAGATG
	Reverse(3′-5′): GGTGAAGGCTCATGTCTGTCTCTG
IL-1β	Forward(5′-3′): GCCCATCCTCTGTGACTC
	Reverse(3′-5′): TGTGCCGTCTTTCATTAC
IFN-γ	Forward(5′-3′): CATTGAAAGCCTAGAAAGTCTG
	Reverse(3′-5′): CTCATGAATGCATCCTTTTTCG
IL-17	Forward(5′-3′): GAGCTTCATCTGTGTCTCTGAT
	Reverse(3′-5′): GCCAAGGGAGTTAAAGACTTTG
IRF4	Forward(5′-3′): AATGGTTGCCAGGTGACAGGAAC
	Reverse(3′-5′): CGCCAAGGCTTCAGCAGACC
STAT3	Forward(5′-3′): GACTGATGAAGAGCTGGCTGACTG
	Reverse(3′-5′): TCCAGACGGTCCAGGCAGATG
IL-4	Forward(5′-3′): GGT CTC AAC CCC CAG CTA GT
	Reverse(3′-5′): TAGTGAACTCTCTCTAGTAGCCG
T-bet	Forward(5′-3′): TCAACCAGCACCAGACAGAG
	Reverse(3′-5′): AAACATCCTGTAATGGCTTGTG
ERα	Forward(5′-3′): ACTGGCCAATCTTTCTCTGC
	Reverse(3′-5′): CAATTCATCCCCAAAGACATGGAC
ERβ	Forward(5′-3′): TCACTTCTGCGCTGTCTGCAGCG
	Reverse(3′-5′): CCTGGGTCGCTGTGCCAAG
GAPDH	Forward(5′-3′): TGCTCGCTGTATTCTTGGTG
	Reverse(3′-5′): GGCTCCTTCTGTCGAGTGAC

### Histological and immunofluorescence detection

Snap-frozen whole TA muscle was transversely cryo-sectioned, with the thickness of 8 μm, and either stained with hematoxylin and eosin or prepared for immunostaining. For immunofluorescence, muscle was fixed with cold acetone and incubated with rat anti-mouse monoclonal antibodies against CD11b (1:200, eBioscience, California, USA), F4/80 (1:200, eBioscience, California, USA), CD4 (1:200, BD or eBioscience, USA), or rabbit polyclonal anti-ERα (1:400, Abcam, USA); mouse monoclonal anti-ERβ (1:200, Abcam, USA), rabbit anti-mouse CD3ɛ (1:200, Abcam, Cambridge, UK), rabbit polyclonal anti-Dystrophin (1:400, Santa Cruz, California, USA), Alexa Fluor 488-conjugated goat anti-rat IgG (1:400, Santa Cruz, California, USA), Alexa Fluor 488-conjugated goat anti-rabbit IgG (1:400, Santa Cruz, California, USA), Cy3-conjugated goat anti-rabbit IgG (1:400, Santa Cruz, California, USA), Cy3-conjugated goat anti-rat IgG (1:400, Santa Cruz, California, USA), Alexa Fluor 555-conjugated donkey anti-mouse IgG (1:500, Beyotime, China), Alexa Fluor 555-conjugated donkey anti-rabbit IgG (1:500, Beyotime, China), FITC-conjugated goat anti-mouse IgG (1:400, eBioscience, USA), or FITC-conjugated goat anti-rabbit IgG (1:400, Santa Cruz, USA) were used as secondary antibodies. Nuclei were counterstained with DAPI. Slides were viewed with an Olympus BX51 fluorescence microscope (Olympus, Japan). For the intensity analysis, the Image-Pro Plus software was performed to quantify the intensity of staining. The integrated optical density (IOD) and area of interest (AOI) of all the positive staining were measured, respectively. The mean density (IOD/AOI) was then calculated.

### Cell sorting and flow cytometry analysis

Damaged TA muscles were collected and minced and then gently digested with 0.2% IIcollagenase at 37 °C for 45 min twice. Total cells isolated from muscle homogenate were re-suspended in fluorescence-activated cell sorting buffer (phosphate buffer solution, 0.5% bovine serum albumin, 2 mM EDTA) to obtain a single-cell suspension. After Fc receptor blocking with anti-CD16/CD32 (Biolegend, USA), cells were incubated with anti-CD45-Pacific Blue, anti-F4/80-PE, anti-Ly-6C-FITC, anti-CD11b-PE, anti-CD3ε-APC, anti-CD4-FITC, anti-IFN-γ-PE, anti-IL-4-APC, anti-IL-17α-PE-Cy7, anti-CD25-PE, anti-Foxp3-APC (1:100, ThermoFisher, USA), anti-T-bet-BV421(1:100, BD Biosciences, USA), anti-CTLA-4-APC (1:100, eBioscience, California, USA), anti-MHC-II-eFluor 450 (1:100, eBioscience, California, USA), and anti-CX3CR1-APC (1:100, Bioss, China). Labeled cells were analyzed with a FACSAria II cell sorter (BD Biosciences, USA) and FlowJo software. For cell sorting, mice were sacrificed on day 3 and 6 after CTX-myoinjury. Muscle samples were collected, minced, and incubated with anti-CD45-Pacific Blue and anti-F4/80-PE on day 3, or incubated with anti-CD3ε-APC and anti-CD4-FITC on day 6. CD45^+^ F4/80^+^ cells, or CD3ε^+^CD4^+^ cells were sorted by MoFlo XDP, for further RNA preparation and qRT-PCR analysis.

### C_2_C_12_ cell culture, proinflammatory stimuli, and the interference with E_2_ or 4-OHT

C_2_C_12_ cells (ATCC, USA) were cultured in Dulbecco’s modified Eagle’s medium Nutrient Mixture F-12 (DMEM/F12, Hyclone) containing with 10% fetal bovine serum (FBS, Gbico), 100 units/ml penicillin, and 100 μg/ml streptomycin sulfate in a 5% CO_2_-humidified chamber (Heraeus, Germany) at 37 °C. For differentiation studies, C_2_C_12_ cells were cultured in DMEM, added with 2% horse serum (Gibco, USA) for 72 h. For proinflammatory stimuli, cells were treated with IFN-γ (0.6 μg/ml, R&D, USA). For in vitro estrogen interference analysis, E_2_ (1.0 nM) or 4-OHT (0.1 μM) [34] were added to the corresponding medium supplemented with IFN-γ, respectively. Cells were analyzed after 48 h culturing with IFN-γ.

### Western blot analysis

Cell or tissue protein extraction was performed according to the manufacturer’s protocol (KeyGEN, China). Protein concentrations were evaluated using a BCA assay kit (KeyGEN, China). Equal amounts of proteins were electrophoresed on 6–12% SDS-polyacrylamide gel and transferred to Immobilon P membrane (Millipore, USA). Membranes were blocked in 5% non-fat dried milk in Tris-buffered saline/Tween-20 (TBS-T: 20 mM Tris, pH 7.5, 150 mM NaCl, 0.05% Tween-20) for 1 h at RT. The following antibodies were used for detection: mouse monoclonal anti-TLR3(1:1000, NOVUS, USA); rabbit polyclonal anti-H-2K^b^ (1:1000, Abcam, Cambridge, UK); mouse monoclonal anti-H2-Ea (1:400, Santa Cruz, USA); rabbit polyclonal anti-ERα (1:1000, Abcam, USA); mouse monoclonal anti-ERβ (1:1000, Abcam, USA); or mouse monoclonal anti-GAPDH (1:4000, KANGCHEN, China). Primary antibodies were incubated for 12 h at 4 °C in 5% non-fat dried milk in TBS-T. The membrane was then washed three times in TBS-T and incubated for 1 h at RT with horseradish peroxidase conjugated goat anti-rabbit IgG (1:5000, Fudebio, China) or goat anti-mouse IgG (1:2000, CST, USA), in 5% non-fat dried milk in TBS-T. After washing three times in TBS-T, the protein bands were visualized by enhanced chemiluminescence (ECL) detection reagents (Applygen Technologic Inc., China). Immunoreactive bands was detected by the ECL detection system (Protein Simple, USA), and densitometric values were analyzed with ImageJ v1.42 software (National Institutes of Health, USA). Relative expression of each immunoreactive band was calculated by comparison with GAPDH.

### ELISA analysis

Mouse blood was collected by retrobulbar vein puncture in the presence of ethylenediamine tetra-acetic acid. Samples were chilled on ice and then plasma-separated by centrifugation at 48 °C for 20 min. Samples was taken for ELISA test of the total estradiol concentration according to the manufacturer’s procedure (the ER EIA kit, R&D, USA). Firstly, the plate was incubated with estradiol primary antibody. Then 100 μl of the standard, control, or samples were added into the appropriate wells on the plate. Further, 50 μl of the estradiol conjugate was then added to all wells and covered with the adhesive strip for 2-h incubation at room temperature on the shaker. After that, each well received 200 μl substrate solution and incubated about 30 min at room temperature on the benchtop, protecting from light. Finally, 100 μl stop solution was added to each well. The optical density of each well was determined within 30 min, using a microplate reader set to 450 nm. The amount of signal was directly proportional to the standard level of estradiol in the assay buffer solution.

### Statistical analysis

All data are expressed as mean ± standard deviation (SD). One-way ANOVA was used for multiple comparisons (Duncan’s multiple range test) using SPSS ver.13.0 software. *P* values < 0.05 were considered as statistically significant.

## Results

### Acute myoinjury induces the enhancement of serum estrogen level and estrogen receptor Erβ expression in damaged muscle and in regenerated myofibres

**Fig. 1:**
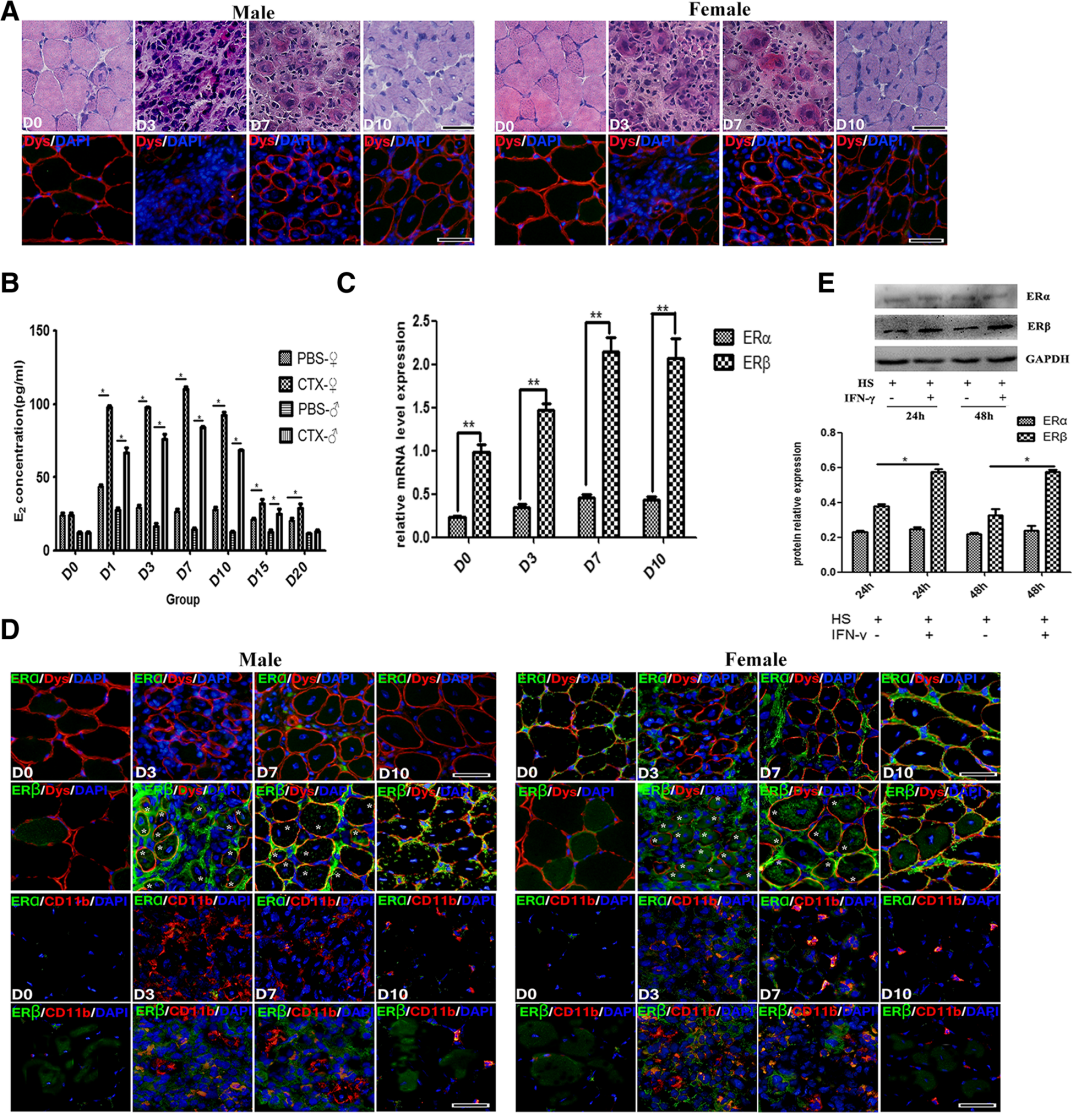
The alternation of serum estrogen level and estrogen receptor expression in CTX-damaged muscle. **a** Histological features of the inflamed TA muscle of male and female mice. The top images: standard H&E staining, and the bottom images: Dystrophin immunofluorescence staining (Red). **b** Elisa assay showing serum E_2_ levels. **c** qRT-PCR analysis presented the mRNA levels of estrogen receptor (ERs) gene in damaged TA muscle. **d** Representative immunofluorescence double-staining results of ERα/ERβ and Dystrophin, or ERα/ERβ and CD11b in damaged TA muscle. Aggregation of ERβ in myofibers was indicated by asterisk (*). **e** Western blots showing protein levels of ERα and ERβ in horse serum-differentiated C_2_C_12_ cells with or without IFN-γ treatment. The relative band intensities from western blots experiments were normalized to the level of GAPDH and analyzed with ImageJ software. All data are presented as mean ± SD (*n* = 3). One-way ANOVA was used for multiple comparisons. (*p < 0.05 and **p < 0.01). *HS* horse serum. Bar = 100 μm

Using H&E and Dystrophin fluorescence staining, we observed that CTX injection in tibialis anterior (TA) muscle of B6 mice induced myofiber necrosis and degeneration at 3 days post-injury. Damage was gradually replaced by smaller regenerating myofibers, and centrally nucleated myofibers became prominent on day 7 and 10 (Fig. 1a). Conspicuous mononuclear cell infiltration was detected on both day 3 and 7 (Fig. 1a).

It has been shown that the serum estradiol (E_2_) level elevated in the early stage of the trauma for the adult male and female patients, which suggests that estrogen may play an important role in protecting vital organs of traumatic patients [17–19]. By means of ELISA assay, we found that serum E_2_ levels significantly increased for male and female mice on day 1, 3, 7, and 10 post-injury (E_2_ peak values presented on day 7), but rapidly decreased on day 15 and 20, compared to that of healthy mice (Fig. 1b), which reflected an early evolving of estrogen signaling in acute injury-induced muscle inflammation procedure. To further establish this point, we turned to qRT-PCR analysis of estrogen receptors (ERs) gene expression in damaged TA muscle, and demonstrated that mRNA levels of estrogen receptor ERα were always lower-expressed after myoinjury, while ERβ gene levels were quite lower in control and 3d damaged muscle, but their levels gradually elevated, and presented peak value at the day 7 and 10 (Fig. 1c), suggesting that ERβ-related estrogen signaling was recruited in response to muscle damage.

We next explored ERα and ERβ expression in damaged muscle through immunofluorescence analysis, and observed that ERα and ERβ low-expressed in mature myofibers from healthy mice (Fig. 1d). However, CTX treatment induced the increased protein expression of ERβ, but not for ERα, in infiltrated mononuclear cells (CD11b^+^) and in regenerated centronuclear myofibers (Dystrophin^+^), as compared to healthy TA muscle (Fig. 1d). To definitely determine whether muscle cells upregulate estrogen signaling in inflammatory milieu, we cultured C_2_C_12_ myoblasts in IFN-γ-constructed proinflammatory milieu, and proved that IFN-γ-induced ERβ protein levels increase in C_2_C_12_ myotubes (2% horse serum administration) (Fig. 1e).

### In vivo estrogen signaling interferes with proinflammatory macrophages recruitment and function after muscle injury

**Fig. 2:**
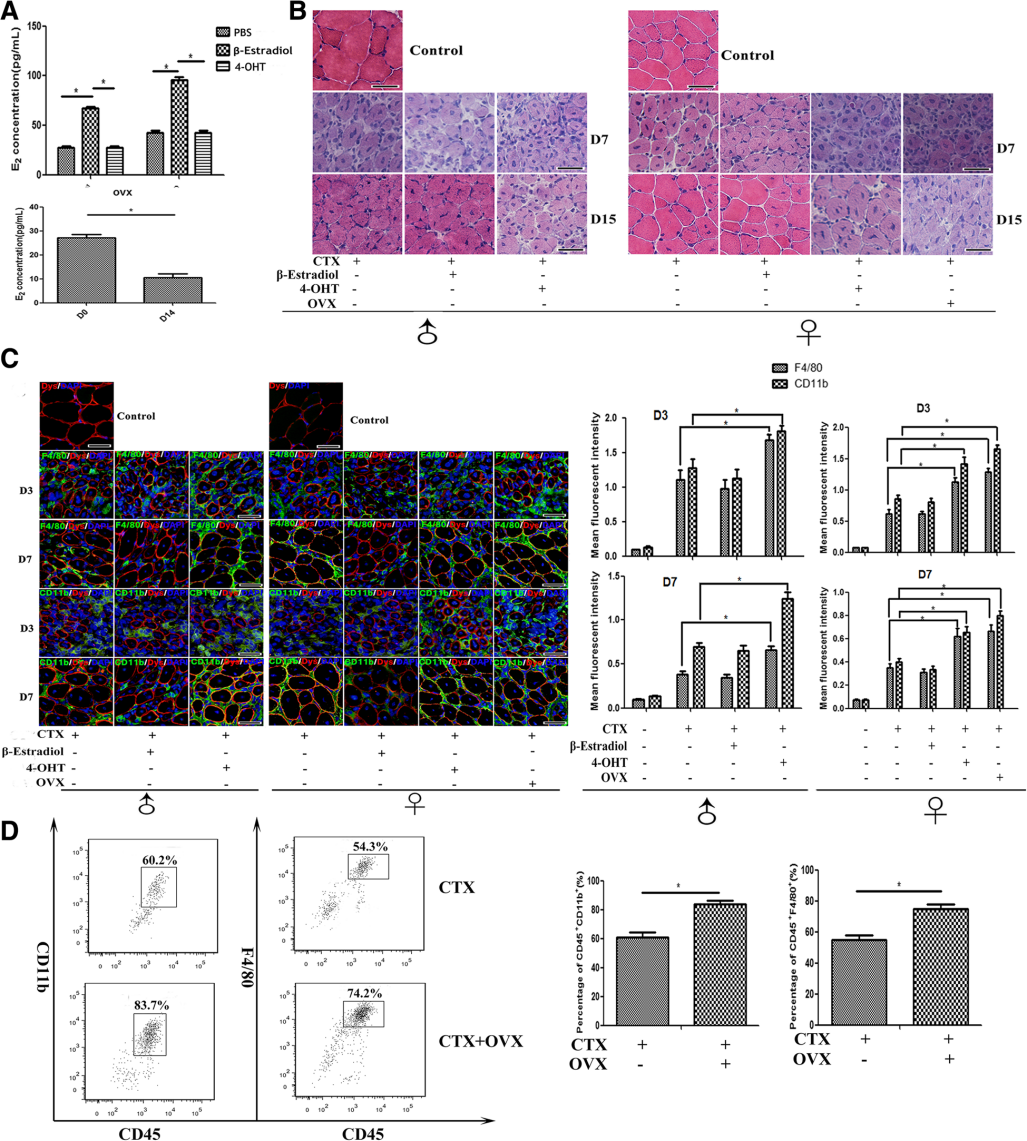
Estrogen signaling interferes with muscle inflammation and regeneration after acute myoinjury. **a** Elisa assay showing serum E_2_ levels in CTX-treated mice received β-estradiol or 4-OHT administration (on day 1 post-injection), or in ovariectomy (OVX) mice. **b** Histological features of damaged TA muscle in CTX-treated mice received β-estradiol or 4-OHT administration, or in CTX-treated OVX mice. **c** Immunofluorescence staining results of CD11b, F4/80 and Dystrophin in damaged TA muscle from B6 mice received β-estradiol or 4-OHT administration, or performed OVX. **d** FACS analysis of the proportion of CD11b^+^ and F4/80^+^ cells in CD45-gated cells isolated from TA muscle on day 3 post-injury in OVX or intact mice. Data are representative of two independent experiments performed with three mice per group. One-way ANOVA was used for multiple comparisons. (*p < 0.05). Bar = 100 μm

Previous studies have shown that estrogen signaling controls crucial processes in immune and inflammatory response [2, 4, 12, 14]. For investigating the in vivo effects of estrogen on muscle inflammation, we next artificially elevated or decreased E_2_ level in vivo, by injecting s.c. of the recombinant β-estradiol, or its antagonist 4-OHT following CTX treatment, respectively. In addition, ovariectomy (OVX) was performed in female mice to get the constant serum E_2_ level decrease in vivo before muscle damage*.* As expectedly, through ELISA test, we confirmed β-estradiol induced serum E_2_ concentration increase in male and female mice separately (Fig. 2a). As reported by Wang Y et al. [35], we found serum E_2_ level of female mice markedly decreased on day 14 post-OVX (Fig. 2a). We did not find the marked difference of muscle inflammation response between β-estradiol administrated and control mice with CTX treatment alone (Fig. 2b); instead, our data indicated that in the absence of estrogen signaling, muscle inflammation was aggravated. Histological analysis showed that 4-OHT injection delayed muscle repair: centrally nucleated myofibers were still prominent on day 15, and muscle architecture was not yet fully repaired at this time point. Indeed, 4-OHT treatment initiated a more sustained and severe inflammatory response in TA muscle, and inflammatory cells were still observed on day 15 post-injury (Fig. 2b). Similar to what was seen in female mice treated with 4-OHT, the delayed muscle repair and the aggravated inflammation were also observed in damaged muscle of female mice that received OVX (Fig. 2b).

Since muscle injury and healing encompass the recruitment of inflammatory cells, and the inflammatory response to acute myoinjury is an innate immune response [36, 37], we next investigated whether estrogen signaling interferes with the intramuscular infiltration of monocytes/macrophages after CTX injection. We conducted immunostaining to assess the infiltration of CD11b^+^ and F4/80^+^ cells in CTX-damaged muscles of B6 mice with or without β-estradiol and 4-OHT treatment, or OVX mice on day 3 and 7 post-injury. The extent of infiltration was evaluated by fluorescent staining intensity analysis. Despite of the similar numbers of inflammatory cells and fluorescence intensity in damaged muscle between β-estradiol-treated and the control mice, we observed a dramatic increase for CD11b^+^ and F4/80^+^ cells in inflamed muscle of mice administrated with 4-OHT, and of OVX mice (Fig. 2c). In line with the immunostaining results, using FACS analysis, we also monitored the increase of CD11b^+^ and F4/80^+^ cells population, in damaged muscle of ovariectomized female mice, compared to control mice (Fig. 2d).

**Fig. 3:**
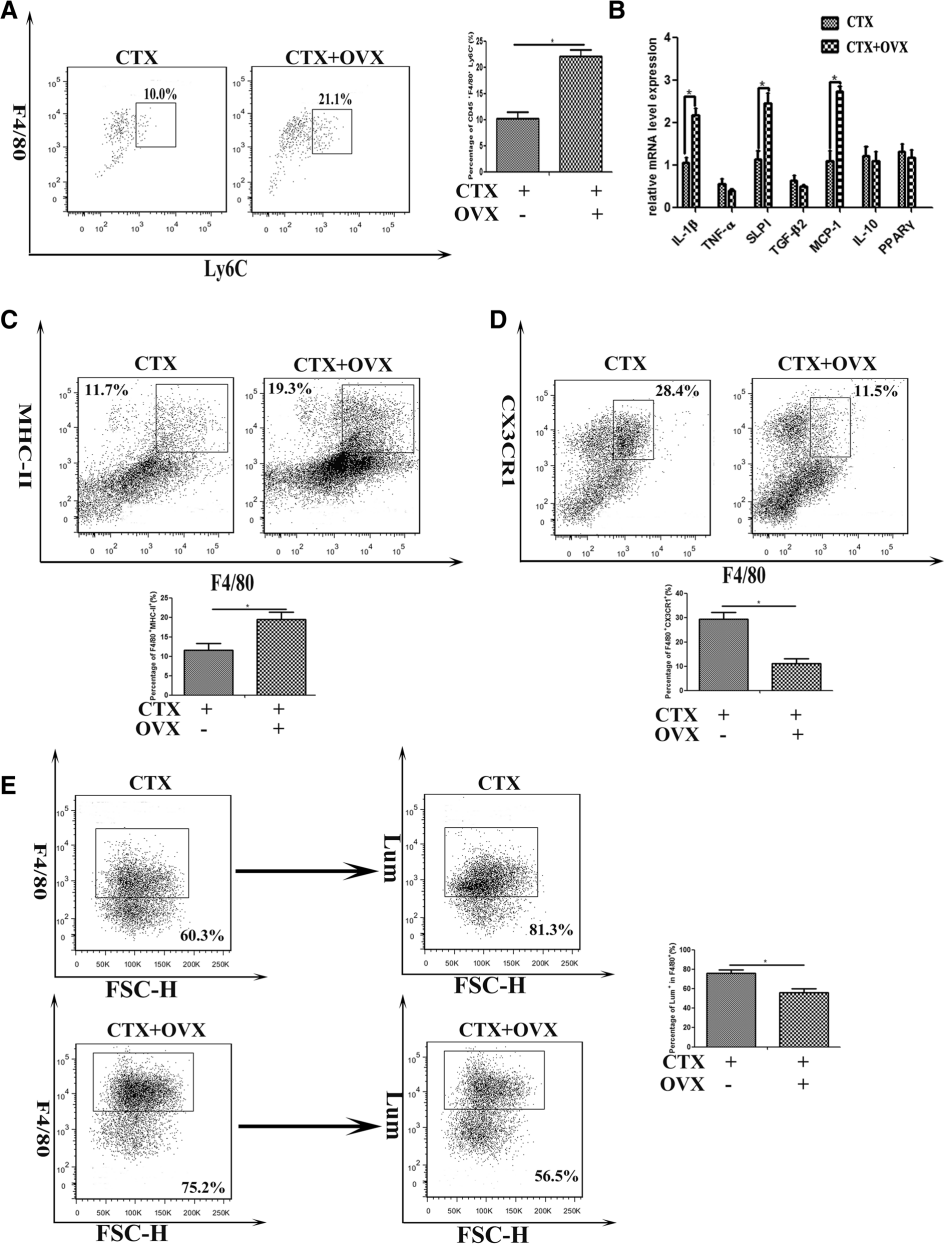
Estrogen signaling effects on the phenotype and function of macrophage infiltrated in inflamed muscle. **a** FACS analysis of the proportion of Ly-6C^+^F4/80^+^ cells in CD45-gated cells isolated from TA muscle on day 3 post-injury, in OVX or intact mice. **b** mRNA levels of IL-1β, SLPI, MCP-1, TNF-α, TGF-β2, IL-10, and PPARγ were quantified by qRT-PCR in CD45^+^F4/80^+^ cells isolated from inflamed muscle on day 3 post-injury in OVX or intact female mice. **c** FACS analysis of the proportion of CD45^+^F4/80^+^MHC-II^+^ cells isolated from inflamed muscle of OVX or intact female mice. **d** FACS analysis of the proportion of CD45^+^F4/80^+^CX3CR1^+^ cells isolated from inflamed muscle of OVX or intact female mice. **e** FACS analysis of the uptake of the injected Lumispheres by CD45^+^F4/80^+^ cells isolated from inflamed muscle of OVX or intact female mice. Data are representative of two independent experiments performed with three mice per group. One-way ANOVA was used for multiple comparisons (*p < 0.05)

To further determine whether estrogen signaling effects on infiltrated macrophage phenotype, CD45^+^ cells were isolated from 3d-damaged TA muscle of female mice receiving ovariectomy or not, labeled with FITC- or PE-conjugated antibodies against Ly-6C or F4/80, and analyzed by FACS. Not surprisingly, our results showed a higher proportion of F4/80^+^Ly-6C^+^ cells in damaged muscle of ovariectomized mice, than that of untreated mice (Fig. 3a). When we turned to address the effects of estrogen signaling on macrophage function, we found that in the absence of estrogen signaling, the gene levels of classical M1-type factors in isolated CD45^+^F4/80^+^ cells, involving IL-1β, SLPI, and MCP-1, but not of M2-type factors (TNF-α, TGF-β2, IL-10, PPARγ), markedly elevated (Fig. 3b). Since MHC-II^+^ macrophages are primarily proinflammatory cells in inflamed muscle, and peaked in number at early phase of regeneration after myoinjury [38], we next monitored MHC-II expression in isolated cells. As presented in Fig. 3c, the higher proportions of F4/80^+^MHC-II^+^ cells were gated from CD45^+^ cells isolated from inflamed muscle of ovariectomized mice, than that of untreated mice. As well, in F4/80^+^ cells from inflamed muscle of ovariectomized mice, we observed the lower expression of CX3CR1, than that of untreated mice (Fig. 3d). These data suggested that estrogen signaling contributes to myoinjury-induced inflammatory infiltration, and maybe favorable for the transition of infiltrated macrophages from proinflammatory M1 to the anti-inflammatory M2 phenotype in damaged muscle.

Previous studies have demonstrated that phagocytic capacity of macrophages is depressed following trauma, whereas administration of estrogen improves macrophage phagocytosis [39]. We next addressed whether estrogen signaling is required for phagocytosis function of macrophage infiltrated in inflamed muscle. For that, CTX myoinjury was performed on OVX mice or untreated control mice, followed by green fluorescent Lum injection into inflamed TA muscle 1d after myoinjury. We found that the uptake of the injected Lum by intramuscular F4/80^+^ cells in OVX mice was 50% lower than that in control mice (Fig. 3e). This data suggested that estrogen signaling may have a role in the macrophages phagocytosis and clearance on dying cells in the damaged/regenerating skeletal muscle.

### Estrogen signaling affects on accumulation and function of CD4 T cells in damaged muscle

**Fig. 4:**
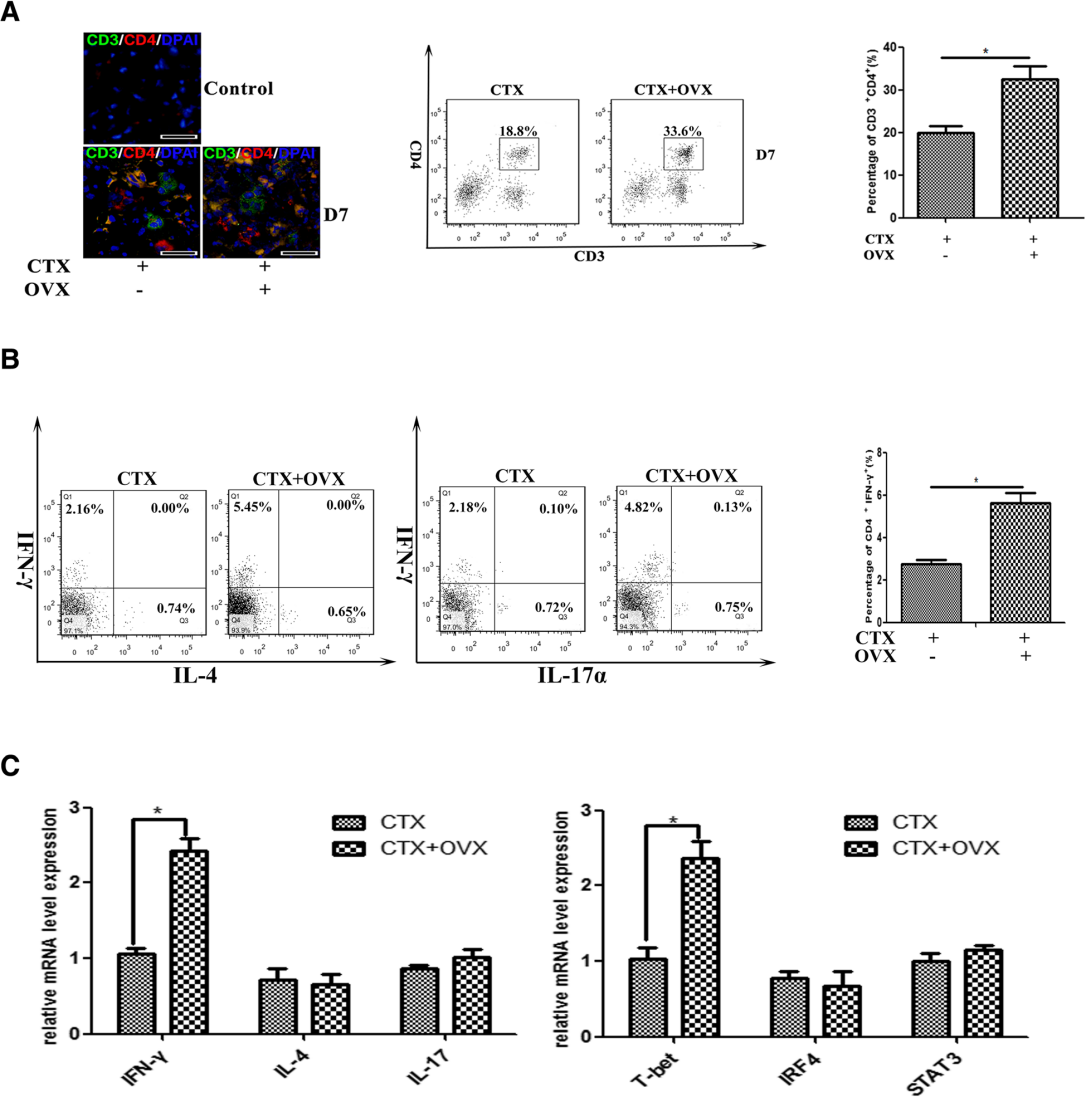
Estrogen signaling affects on accumulation and function of CD4^+^ cells in damaged muscle. **a** Immunofluorescence double-staining (left) and FACS analysis (right) of CD4^+^ T cells in inflamed muscle. **b** FACS analysis of the frequency of Th1(IFN-γ^+^), Th2(IL-4^+^), and Th17(IL-17^+^) cells gated from CD4^+^ cells isolated from 7d-damaged TA muscle, in OVX or intact female mice. **c** mRNA levels of cytokine IFN-γ, IL-4, IL-17 and transcription factor T-bet, IRF4, STAT3 in CD3^+^CD4^+^ T cells isolated from 6d-damaged TA muscle in OVX or intact female mice, were quantified by qRT-PCR. Data are representative of two independent experiments performed with three mice per group. One-way ANOVA was used for multiple comparisons (*p < 0.05). Bar = 50 μm

Muscle antigens have been shown to induce migration of T lymphocytes to the endomysium or perimysium after myoinjury [40, 41]. To probe whether estrogen signaling is involved in the intramuscular T cell infiltration, we conducted double immunostaining to assess the presence of CD8^+^ T cells (CD3ε^+^CD8α^+^) and CD4^+^ T cells (CD3ε^+^CD4^+^) in CTX-damaged muscle of ovariectomized female mice. Unexpectedly, we did not find a remarkable number difference of CD8^+^ T cell in damaged muscle between OVX and control mice (data not shown). Instead, following ovariectomy, an increased ratio of CD4^+^ T cells were found in damaged muscle (Fig. 4a). To further ascertain whether estrogen signaling affects on intramuscular CD4^+^ T cell phenotypes, we examined the frequency of T helper-1 (Th1), T helper-2 (Th2), and T helper-17 (Th17) cells among muscle-infiltrated CD4^+^ T cell, by the FACS analyzing of the expression of IFN-γ, IL-4, and IL-17 in those cells. Our results showed that the frequency of IFN-γ^+^CD4^+^ cells was significantly increased in the inflamed muscle of ovariectomized mice compared to controls (Fig. 4b), suggesting that estrogen signaling is required for suppressing Th1 response in damaged muscle. To definitively establish this point, we isolated CD3^+^CD4^+^ T cells from 6d-damaged TA muscle of female mice that received OVX or not, and examined gene levels of cytokine IFN-γ, IL-4, and IL-17, as well the levels of the Th1-specifying transcription factor T-bet, Th2 factor IRF4, and Th17 factor STAT3 by qRT-PCR analysis. In line with the FACS results, the increased mRNA expression of IFN-γ and T-bet was seen in CD4^+^ T cells isolated from inflamed muscle of OVX mice, comparing to that of control mice (Fig. 4c).

**Fig. 5:**
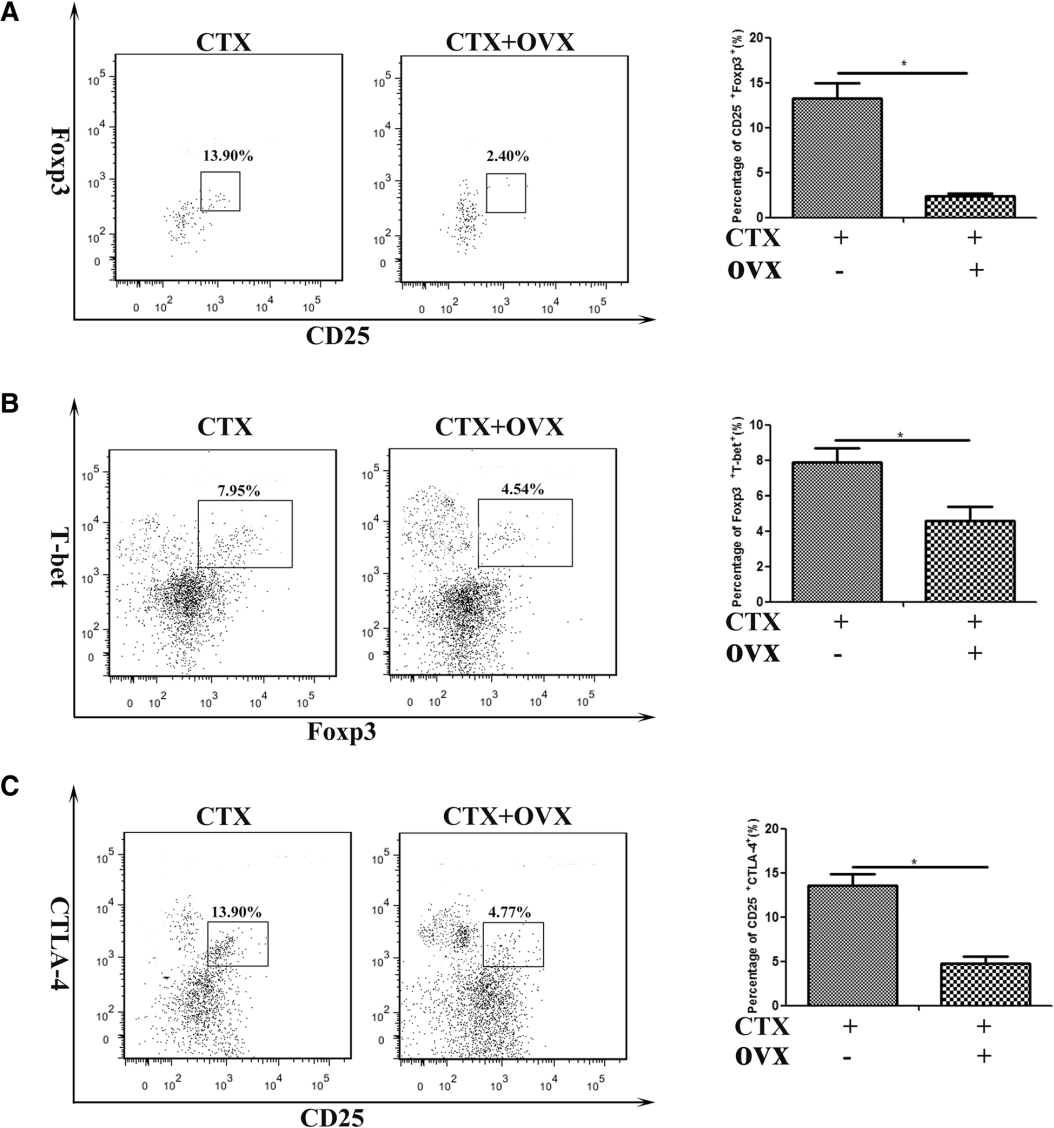
Estrogen signaling affects on accumulation and function of Treg cells in damaged muscle. FACS analysis of the frequency of the CD4^+^CD25^+^Foxp3^+^ cells (**a**), CD4^+^T-bet^+^Foxp3^+^ cells (**b**), and CD4^+^CD25^+^CTLA-4^+^ cells (**c**) isolated from 7d-damaged TA muscle in OVX or intact female mice. Data are representative of two independent experiments performed with three mice per group. One-way ANOVA was used for multiple comparisons (*p < 0.05)

Acute myoinjury provokes the local accumulation of a special population of CD4^+^Foxp3^+^ regulatory T cells (Tregs) within days, which exerts immunosuppressive actions, and promotes muscle repair [42, 43]. Some findings reveal a role for a Foxp3-dependent mechanism of E_2_ control of peripheral T cell tolerance [44]. To determine whether estrogen signaling affects on Tregs infiltration and function, we investigated Tregs accumulation in CTX-damaged TA muscle from female mice received ovariectomy or not, on day 6 post-myoinjury. Interestingly, despite the increase of CD4^+^ T cell population in damaged muscle of ovariectomized mice (Fig. 4a), we monitored a significant decrease for the percentage of CD4^+^CD25^+^Foxp3^+^ T cells in inflamed muscle (Fig. 5a). Given the reported roles of Tregs in skeletal muscle repair [42, 43], we suppose myofiber regeneration delay in 4-OHT administered or ovariectomized mice may be partly reasoned by the loss of estrogen signaling restrained Tregs accumulation in damaged muscle.

Previous study has shown that T-bet promotes expression of the chemokine receptor CXCR3 on Treg cells, and T-bet^+^ Treg cells accumulated at sites of Th1-mediated inflammation [45]. Since we monitored the elevation of Th1 response in damaged muscle of ovariectomized mice, we next addressed T-bet expression in muscle-infiltrated Treg cells. We exhibited that in the absence of estrogen signaling, T-bet^+^Foxp3^−^ cells significantly increased in inflamed muscle, but T-bet^+^Foxp3^+^ cells markedly reduced (Fig. 5b). In order to evaluate the effect of estrogen signaling on Treg cell function, we examined the expression of CTLA-4, which is required for Treg suppressive function [46], on CD4^+^CD25^+^ cells gated from inflamed muscle. Not surprisingly, we observed the significantly decreased frequency of CD25^+^CTLA-4^+^ cells in the inflamed muscle of OVX mice, comparing to that of control mice (Fig. 5c). Thus our data outline that after acute damage, the upregulated estrogen signaling prompts to Treg cell accumulation in damaged muscle, which is favorable for suppressing of Th1 responses, and for promoting to muscle regeneration.

### Estrogen signaling affects on intrinsic immunological behaviors of muscle cells

**Fig. 6:**
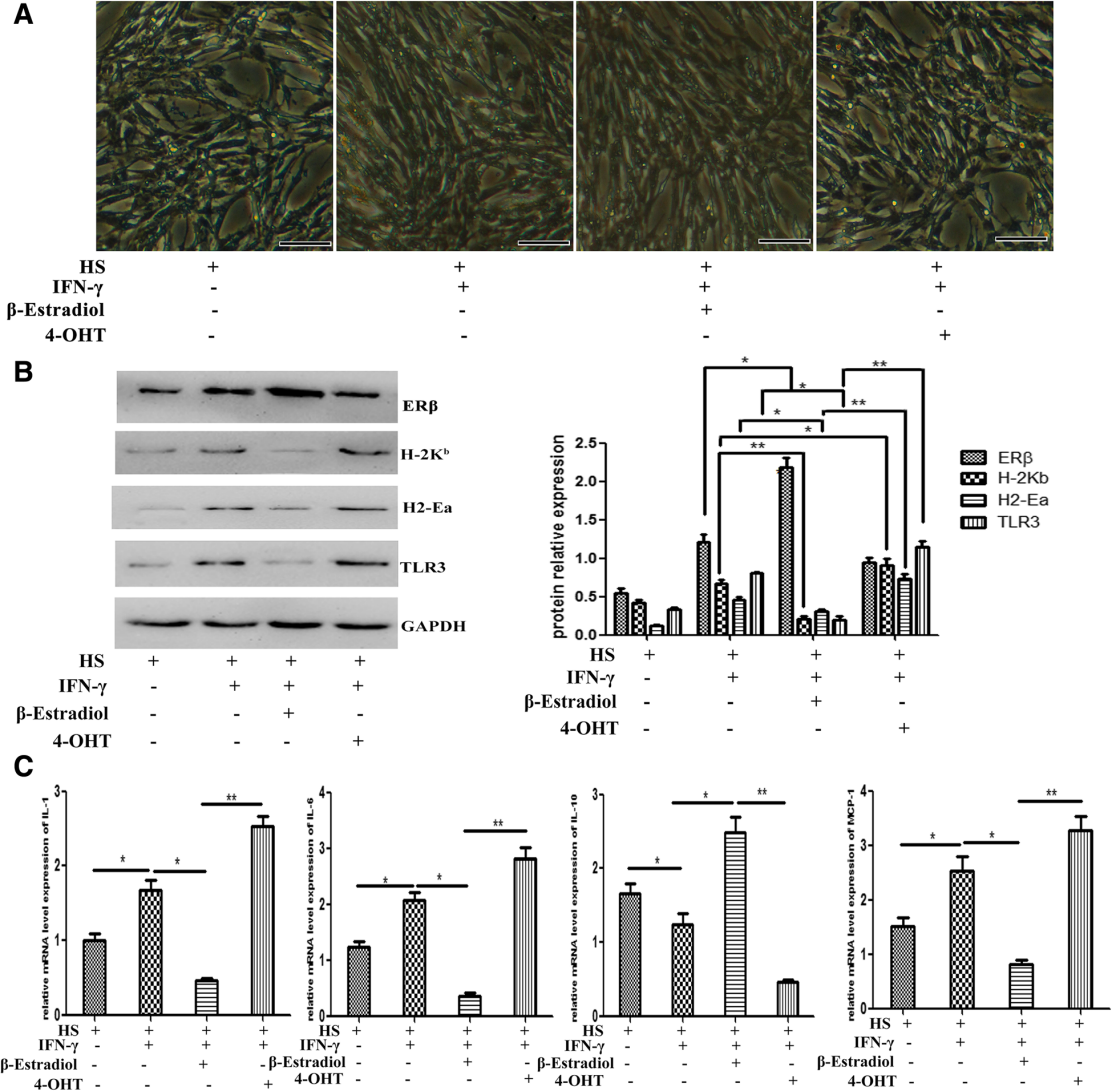
Estrogen signaling affects on intrinsic immunological behaviors of muscle cells. **a** Phase contrast microscopical observation demonstrated C_2_C_12_ cells survived and differentiated (2% horse serum) well in IFN-γ-, E_2_-, or 4-OHT-added medium. **b** Western blots analysis showed protein level changes of H-2K^b^, H2-Ea, TLR3, and ERβ in HS-differentiated C_2_C_12_ myotubes, in the presence of IFN-γ, E_2_, or 4-OHT. The relative band intensities from western blots experiments were normalized to the level of GAPDH and analyzed with ImageJ software. **c** mRNA levels of IL-1, IL-6, MCP-1, and IL-10 were quantified by qRT-PCR in C_2_C_12_ myotubes, in the presence of IFN-γ, E_2_, or 4-OHT. All data are presented as mean ± SD (*n* = 3). One-way ANOVA was used for multiple comparisons. (*p < 0.05 and **p < 0.01). *HS* horse serum. Bar = 50 μm

Myoblasts and myotubes can express a surprising variety of immunologically important molecules, including class I/II major histocompatibility complex (MHC-I/II) and co-stimulatory molecules in the presence of IFN-γ or other proinflammatory cytokines [47, 48]; this suggests that, under proinflammatory environment, muscle cells can actively participate in local immune reactions. We have demonstrated that estrogen signaling contributed to muscle inflammation response, which could reflect that estrogen signaling possibly affects on intrinsic immunological capacities of muscle cells, like it does for immune cells. To clarify this point, we next evaluated the effects of estrogen signaling on immunological behaviors of muscle cells induced by IFN-γ. For that, cultured and then horse serum-differentiated C_2_C_12_ cells were treated with β-estradiol or 4-OHT, separately. We found that C_2_C_12_ cells survived well in β-estradiol- or 4-OHT-added medium, and can be successfully induced to form multinucleated myotubes under horse serum administration (Fig. 6a). In this experimental setting, differentiated myotubes upregulated protein levels of MHC-I molecule H-2K^b^, MHC-II molecule H2-Ea, and TLR3 under IFN-γ stimulation (Fig. 6b), which imply that muscle cells gain properties relevant for driving the immune response under inflammatory environment. Consistently, we found that C_2_C_12_ myotubes received IFN-γ stimuli upregulated protein level of ERβ, and the adding of β-estradiol further enhanced ERβ level (Fig. 6b). In β-estradiol-treated myotubes, we detected a significant protein level downregulation of H-2K^b^, H2-Ea, and TLR3 under IFN-γ stimulation. Instead, 4-OHT induced a remarkable upregulation for these molecules (Fig. 6b).

The final detrimental or beneficial effect of the inflammatory response on muscle repair is influenced by specific interactions between inflammatory mediators that act as positive and/or negative regulators to coordinate local and systemic inflammatory-related events and modulate muscle repair process [49, 50]. To evaluate more directly how immunological capacities of muscle cells is affected by estrogen signaling, we next analyzed the expression of the selected proinflammatory cytokines involving IL-1 and IL-6, anti-inflammatory cytokines involving TGF-β and IL-10, and the macrophage-sourced chemokines involving MCP1 and MIP-1α, which have been associated with the preferential biological role during the muscle damage-induced inflammation response [51], in differentiated C_2_C_12_ cells treated with β-estradiol- or 4-OHT, separately. Our qPCR analysis demonstrated, in the presence of IFN-γ, that IL-10 expression was significantly inhibited by 4-OHT administration, but promoted by β-estradiol (Fig. 6c). β-estradiol treatment resulted in a significant mRNA levels downregulation of proinflammatory IL-1 and IL-6 (Fig. 6c). In contrast, 4-OHT stimulated a conspicuous increase of these two cytokines in myotubes (Fig. 6c). Similarly to what was observed above, we noticed that after changing of estrogen level artificially in vitro, the expression of macrophage-sourced chemokines in muscle cells was altered: β-estradiol treatment effectively diminished MCP-1 levels, but not for MIP-1α. Conversely, 4-OHT induced a striking increase in the production of MCP-1 (Fig. 6c). Taken together, these results collectively suggest that estrogen signaling is crucial for regulating the intrinsic immune behaviors of muscle cells.

## Discussion

In the clinic, it was noted that after the acute trauma, the females were comparatively insensitive to the injury and had better function recovery than the male. This sexual difference in the damaged tissue or organs protection effect was related with the sex hormone levels in the blood circulation and their possible effects on traumatic insult [15–19]. There is evidence showing the serum E_2_ level significantly increased in the early stage in male and female acute traumatic patients [15, 20]. While, recent studies indicated that estrogen influences the immunologic responses to traumatic insult in animals. Claudia H. Tambeli et al. proved that estradiol administration in ovariectomized female mice significantly decreased formalin-induced plasma extravasation and neutrophil migration in the temporomandibular joint region, an effect that was blocked by the estrogen receptor antagonist ICI 182780 [52]. Mukai K. reported that estrogen therapy can reduce the number of wound neutrophils, and reduce the neutrophil adhesion molecule l-selectin, leading to diminished neutrophil localization at sites of inflammation [53]. Using CTX-induced muscle damage model, we monitored a significant elevation for serum E_2_ levels in male and female mice at the early stage, but a rapid decrease at the later stage post-injury reflected an early evolving of estrogen signaling in acute muscle injury procedure. By immunostaining and protein level analysis, we observed the increased ERβ expression in infiltrated inflammatory cells, and in regenerated myofibers. Previous reports suggested that estrogen is required for mediating glucose up-take in skeletal muscle, and for the activation of myogenic precursor cells in response to a myotrauma [30, 31]; here, we further enriched the issue for its role on muscle inflammation response after myotrauma.

Skeletal muscle is a target tissue for estrogen. Several muscle pathologies are caused, in part, by decreased estrogen levels [29]. In inflamed muscle, neutrophil invasion is exacerbated by ovariectomy (OVX) and attenuated by estrogen treatment, while ER antagonist will attenuate effects of estrogens on leukocyte infiltration in damaged muscle [54, 55]. E_2_ is further suggested to reduce inflammatory gene expression in macrophages by inhibiting nuclear factor kappa-light-chain-enhancer of activated B cells (NF-κB) intracellular transport [56]. Our data demonstrated that 4-OHT injection and OVX treatment delayed muscle repair, and initiated a more severe monocytes/macrophages infiltration in damaged TA muscle. Our data thus highlight the ability of estrogen to prevent secondary muscle damage by modulating inflammatory dynamics of skeletal muscle after injury. During the inflammatory phase of wound healing, estrogen have been shown to promote alternative macrophage polarization (promote a shift from M1 to M2 subtypes), and thus reduce the expression of proinflammatory cytokines [53, 56]. Consistently, we monitored the frequency increase of M1 macrophages and the elevation of inflammatory molecules IL-1β, SLPI, and MCP-1 levels in macrophages isolated from damaged muscle of OVX mice, supporting the important roles of estrogen-mediated signaling transduction in dampening muscle inflammation procedure. In addition, we observed that in damaged muscle, macrophage phagocytosis was suppressed following OVX treatment. This is consistent with the previous studies demonstrating that estrogen administration restored liver macrophages phagocytic capacity following trauma-hemorrhage [39], and suggested the role of estrogen signaling in mediating muscle inflammation response and repair.

The mechanism by which estrogen affects T cell biology has not yet been fully elucidated, and the overall picture is complicated by what appears to be a biphasic response to estrogen by T cells. Indeed, low estrogen states skew the T-helper (Th) response toward a Th1 polarization with associated increased cell-mediated immunity, whereas high doses of estrogen unbalance the Th cell differentiation toward a Th2 phenotype and associated humoral responses [57]. We explored this issue in damaged muscle, and outline that estrogen signaling is key to suppress intramuscular accumulation of CD4^+^ T cells. In addition, we noticed that in the absence of estrogen signaling, the Th cell differentiation toward a Th1 phenotype in damaged muscle. In female, Treg cells frequency and number increase during the follicular phase due to the increase in estrogen levels, while they decrease during the luteal phase when estrogen is low [9]. ER deletion in T cells contributed to the reduction of T cell activation and proliferation and increasing the expression of *Foxp3*, which encodes a critical transcription factor for the differentiation and function of regulatory T cells. Under iTreg conditions, the addition of E_2_ enhanced the frequency of Foxp3^+^ cells [58]. Our study suggests that in inflamed muscle, the accumulation and function of Treg cells depends on estrogen level, at least in part, which is favorable for Th1 responses suppression. It has been reported that after acute myoinjury, Treg cells dampens proinflammatory macrophages by reigning in a local IFN-γ response [38]. Therefore, according to our results, we could speculate that in inflamed muscle, estrogen signaling dampens proinflammatory macrophages by increasing the Treg cells induced-Th1 response suppression.

Estrogen has been shown to regulate terminal differentiation and survival of antigen-presenting cell (APCs). Seillet et al. reported the effects of 17*β*-estradiol on the functional response of steady-state and activated DCs. Specifically, E_2_ boosts the differentiation and effector functions of inflammatory DCs derived from GM-CSF-stimulated myeloid precursors, through IFN regulatory factor (IRF)-4 upregulation [59]. This finding aligns with previous studies where it was shown that E_2_ potentiates and sustains GM-CSF induction of IRF4 in vitro and in vivo [60]. Estrogens also affect the behavior of mature DCs. The study by Siracusa et al. reported that DCs exposed to estrogens in vitro displayed an increase in IFN*γ* ^+^killer DCs (CD11c^+^MHC class II^+^CD49b^+^Nk1.1^high^ phenotype) [61]. Skeletal muscle cells have been proposed to be the facultative APCs. Cultured human myoblasts express constitutively the classical human leukocyte (HLA) class I antigens, TLR3 and TLR7, and the expression level is increased by proinflammatory cytokines (e.g., IFN-γ, TNF-α, IL-1α, etc.), which hints that muscle cells can actively participate in local immune reactions [47, 48]. In mice, ERβ is the predominant ER isoform [28]. In agreement, we detected the increased ERβ expression in inflamed muscle tissue and in regenerated myofibers. In vitro, we observed the obvious expression upregulation of H-2K^b^, TLR3, and H2-Ea in the differentiated C_2_C_12_ myotubes in the presence of proinflammatory IFN-γ. Moreover, we provide evidence for a suppressive role of estrogen signaling in the intrinsic immune behaviors of muscle cells, since we detected a significant downregulation of H-2K^b^, H2-Ea, and TLR3 levels and some proinflammatory cytokines in β-Estradiol-treated C_2_C_12_ myotubes, but a remarkable upregulation in 4-OHT-treated myotubes, under IFN-γ stimulation.

## Conclusions

Collectively, our data suggest that estrogen is a positive intervention factor for muscle inflammatory response. In vivo, estrogen devotes to reduce inflammatory damage of skeletal muscle, through its effects on controlling intramuscular infiltration of monocytes/macrophages and the transition of proinflammatory M1 to the anti-inflammatory M2 macrophages, affecting accumulation and function of Treg cells, and suppressing Th1 response in damaged muscle. Our findings also imply an inhibition effect of estrogen on the intrinsic immune behaviors of muscle cells.

## Abbreviations

4-OHT: 4-Hydroxytamoxifen
APC: Antigen-presenting cell
CTX: Cardiotoxin
DC: Dendritic cell
DMEM: Dulbecco’s modified Eagle’s medium
E2: Estradiol
ERs: Estrogen receptors
FBS: Fetal bovine serum
GAPDH: Glyceraldehyde-3-phosphate dehydrogenase
GM-CSF: Granulocyte-macrophage colony stimulating factor
HLA: Human leukocyte
HS: Horse serum
IFN-γ: Interferon-γ
IRF: Interferon regulatory factor
Lum: Lumispheres
MHC-I/II: Class I/II major histocompatibility complex
NF-κB: Nuclear factor kappa-light-chain-enhancer of activated B cells
NK: Natural killer
OVX: Ovariectomy
TA: Tibialis anterior
TGF-β: Transforming growth factor-β
TLR: Toll-like receptors
