# Correction: Estrogen signaling effects on muscle-specific immune responses through controlling the recruitment and function of macrophages and T cells

**DOI:** 10.1186/s13395-022-00298-5

**Published:** 2022-06-24

**Authors:** Zhao Hong Liao, Tao Huang, Jiang Wei Xiao, Rui Cai Gu, Jun Ouyang, Gang Wu, Hua Liao

**Affiliations:** 1 Guangdong Provincial Key Laboratory of Medical Biomechanics, Department of Anatomy, School of Basic Medical Sciences, Southern Medical University, Guangzhou, 510515 China; 2 Department of Emergency, NanFang Hospital, Southern Medical University, Guangzhou, 510515 China

**Correction: Skeletal Muscle 9, 20 (2019)**

**https://doi.org/10.1186/s13395-019-0205-2**

Following publication of the original article [1], the authors subsequently identified an error in the affiliation address of the first author (Zhao Hong Liao). The corrected affiliation is shown below.

^1^Guangdong Provincial Key Laboratory of Medical Biomechanics, Department of Anatomy, School of Basic Medical Sciences, Southern Medical University, Guangzhou
